# A Five-Electrode Contactless Conductivity Detector Based on a Sandwiched Microfluidic Chip for Miniaturized Ion Chromatography

**DOI:** 10.3390/s26010089

**Published:** 2025-12-23

**Authors:** Kai Chen, Ruirong Zhang, Mengbo Wang, Bo Wang, Shaoshuai Wang, Haitao Zhao

**Affiliations:** Ministry of Education Key Laboratory of Micro/Nano Systems for Aerospace, Key Laboratory of Micro- and Nano-Electro-Mechanical Systems of Shaanxi Province, School of Mechanical Engineering, Northwestern Polytechnical University, Xi’an 710072, China; chnek@mail.nwpu.edu.cn (K.C.); wmb_zhk@163.com (M.W.); wbo@nwpu.mail.nwpu.edu.cn (B.W.); shaoshuaiwang@mail.nwpu.edu.cn (S.W.)

**Keywords:** contactless conductivity detection, microfluidic, ion chromatography (IC), chromatography chip, miniaturized detector

## Abstract

This study aims to develop a chip-based five-electrode contactless conductivity detector for miniaturized ion chromatography (IC) systems. The detector comprises a detection chip (50 mm × 25 mm × 6 mm) and a detection circuit. The detection chip consists of a top layer, an insulating film, and a bottom layer wherein a planar five-electrode printed circuit board (PCB) is embedded. Among the five electrodes, one shielding electrode is designed to suppress the leakage current in the flow channel; consequently, the potential at the solution outlet is raised, further enhancing detection sensitivity. Furthermore, integrating the electrodes into a PCB module can reduce the difficulty of electrode fabrication and extend the lifespan of the electrodes. The detector was applied to a commercial IC system and successfully achieved the separation and detection of three anions (Cl^−^, NO_3_^−^, SO_4_^2−^). For standard solutions, the limit of detection (LOD) values of Cl^−^, NO_3_^−^ and SO_4_^2−^ are 0.47, 0.80, and 0.95 ppm, respectively. For mixed samples, the separation analysis was completed within 25 min, and the maximum detection error is no more than 2.2%. The five-electrode contactless detector developed shows great potential for application in miniaturized ion chromatography.

## 1. Introduction

Ion Chromatography (IC) is an efficient analytical technique for separating and detecting ions and polar molecules in solutions, and it has been widely applied in chemical analysis, water quality monitoring, environmental science, and other related fields [1,2,3]. To respond to the requirements for on-site analysis and real-time sample detection, IC systems are exhibiting an increasingly prominent development trend toward miniaturization and portability [4,5,6,7]. Within this trend, the core of IC system miniaturization lies in the miniaturization of their key modules, among which the miniaturization of detectors is particularly critical [8,9]. Common detectors in current IC systems include UV–visible (UV–vis) absorbance detectors, mass spectrometers (MS), and conductivity detectors. Among these, the miniaturized UV–vis absorbance detectors usually suffer from a low sensitivity because their effective optical path has been significantly reduced [10]. MS are characterized by complex structures and relatively high costs, which have resulted in their application in miniaturized IC systems remaining a considerable challenge [11]. In contrast, conductivity detectors offer excellent sensitivity for ionic species and can detect trace amounts of analytes. Additionally, conductivity detectors feature simple working principles, making them easier to integrate into miniaturized devices, and these characteristics make them more promising for miniaturized IC systems [12,13]. Conductivity detectors can be performed in contact and contactless modes. Early conductivity detection primarily used the contact mode [14,15]. The direct contact of electrodes with the solution tends to trigger electrochemical erosion on the electrode surface, resulting in detrimental effects [16,17]. In the contactless mode, electrodes do not directly contact the measured fluid. An insulating layer is used here to physically isolate electrodes from the sample solution. This design effectively avoids the drawbacks of the polarization effect and electrochemical erosion [17,18]. Additionally, contactless conductivity detectors have simple working principles and configurations, facilitating the miniaturization of the devices [19].

Currently, constructing contactless conductivity detection cells on microfluidic chips is the primary approach to miniaturizing contactless conductivity detectors [17]. However, the insulating layer integrated on the chip introduces an equivalent capacitance, which significantly reduces the coupling efficiency between electrodes and the solution. Consequently, this results in low sensitivity [17,20]. To tackle this issue, researchers have primarily explored two strategies [21,22,23,24,25,26,27,28,29]. The first strategy involves optimizing detection principles, utilizing advanced configurations such as four-electrode method [21] to minimize the equivalent impedance of the insulating layer. The second strategy focuses on innovating detection chip structures or fabrication processes, for instance, by adopting a dual-channel design [26] or an insulating layer with controllable thickness [29] to enhance the performance of the detector. Although these strategies have to some extent enhanced the performance of the detector, they exhibit inherent limitations. Principle-based optimizations often overlook the impact of leakage current in the flow channel, and framework-based innovations usually introduce significant fabrication complexity and higher production costs. Therefore, there are significant challenges in developing high-sensitivity contactless conductivity detectors.

To address the aforementioned issues, this study proposes a contactless conductivity detector based on a five-electrode chip. By adopting a five-electrode configuration, a voltage detection path with high input impedance is introduced, which can effectively eliminate the influence of the insulating layer capacitive impedance on the detection circuit. Additionally, the shielding electrode was placed on the solution outlet side of the detection cell, and the shielding voltage was applied to this electrode to raise the potential at the solution end. This can effectively suppress loop current leakage, enabling a high-sensitivity conductivity detection at relatively low operating frequencies. The detection cell chip with a sandwich structure was designed. In the hollowed-out region of the bottom chip, the PCB five-electrode module was embedded, with its sensing surface closely attached to the insulating film. This design not only maintains excellent detection performance but also significantly reduces the structural complexity and fabrication cost of the detection cell chip. This detector exhibited a good linear response to the standard solutions of NaCl, NaNO_3_, and Na_2_SO_4_. After integrating the miniaturized chip detector with a commercial ion chromatography separation device, the assembled system was utilized to analyze the concentration levels of three anions (Cl^−^, NO_3_^−^, SO_4_^2−^). The results indicate that the five-electrode contactless conductivity detector has been successfully applied to the IC system, demonstrating considerable potential for application in the development of miniaturized chromatographs.

## 2. Materials and Methods

### 2.1. Reagent

Sodium chloride was acquired from Sigma-Aldrich Trading Co., Ltd. (Shanghai, China); sodium bicarbonate, sodium carbonate, sodium nitrate, and sodium sulfate were obtained from Adamas Reagent Co., Ltd. (Shanghai, China), with all reagents of analytical grade (AR). Purified water (resistivity > 18.2 MΩ) was used to prepare all solutions. Clearweld laser welding coatings (LD940) were purchased from Crysta-lyn Chemical Co. (Endicott, NY, USA).

### 2.2. Detection Principle

**Figure 1:**
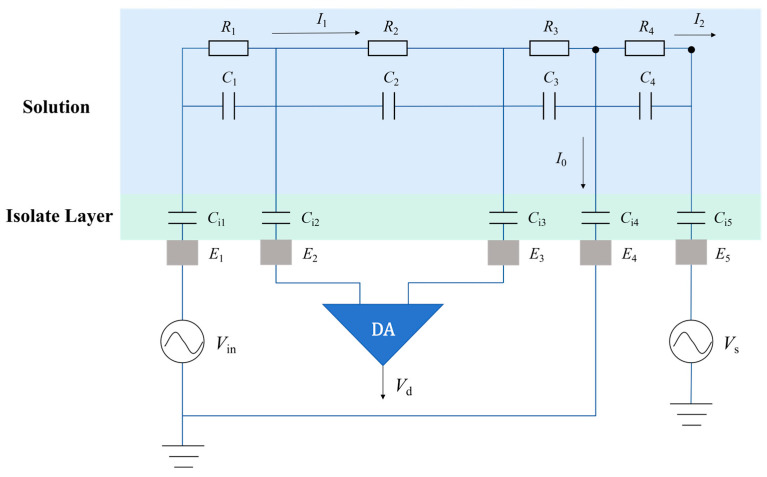
The equivalent circuit model of the sandwiched detection chip (a differential amplifier is externally connected to $E_{2}$ and $E_{3}$).

The detection principle adopted in this study is developed based on the four-electrode contactless conductivity detection method proposed by Laugere et al. [21]. In this method, the configuration was designed with a five-electrode structure, which includes 1 excitation electrode (*E*_1_), 2 voltage output electrodes $(E_{2}$ and $E_{3}$), 1 current output electrode ($E_{4}$), and 1 shielding electrode $\left(E_{5}\right)$. In Figure 1, $C_{1}{\sim C}_{4}$ is the equivalent capacitance of the solution, the solution impedance consists of a parallel combination of solution resistance (R) and solution capacitance (C), $C_{i}$ is the equivalent capacitance of the insulating layer between each electrode and the solution, $I_{1}$ is the actual current flowing through the solution to be tested, $I_{0}$ is the output current, and $I_{2}$ is the flow path leakage current.

By connecting electrodes $E_{2}$ and $E_{3}$ to a high-input-impedance differential amplifier, the current flowing through the voltage detection loop becomes very small and can be neglected. This design can effectively prevent the voltage output electrodes ($E_{2}$ and $E_{3}$) from shunting current from the main loop ($E_{1}$ and $E_{4}$), thereby maintaining the integrity of the current detection loop. Meanwhile, in accordance with Ohm’s law, the negligible current ensures that the voltage drop across the insulating layer capacitors ($C_{i2}$ and $C_{i3}$) is insignificant. Consequently, the potential difference measured between $E_{2}$ and $E_{3}$ accurately reflects the true ohmic voltage drop across the solution resistance ($R_{2}$), effectively eliminating the interference of the insulating layer’s impedance [21]. In addition, applying a shielding voltage of a specific amplitude to the shielding electrode can effectively suppress the leakage current in the flow path of the detection cell. By applying an appropriate peak value of the shielding voltage, the output current ($I_{0}$) of the current detection electrode $\left(E_{4}\right)$ is approximately equal to the actual current ($I_{1})$ flowing through the solution to be tested. For ease of analysis and calculation, non-ideal factors with minimal impact and difficult quantitative analysis, including leakage current, inter-electrode stray capacitance, Zeta potential, and flow channel internal surface resistance, are ignored [30].

According to the simplified equivalent circuit model, the total loop impedance $(Z_{total}$) is jointly determined by the solution impedance (Z) and the equivalent capacitance impedance of the insulating layer $\left(Z_{i}\right)$, where the solution impedance consists of a parallel combination of the equivalent solution resistance (R) and the equivalent solution capacitance (C) [30]. Under small-signal excitation conditions, this complex non-linear system can be validly simplified into a linear circuit model [31]. Consequently, the impedance components can be expressed as

(1) $$Z=R\;//\;C=\frac{R}{RjwC+1}$$

(2) $$Z_{i}=-\frac{1}{jwC_{i}}$$

(3) $$Z_{total}=Z_{ci1}+Z_{1}+Z_{2}+Z_{3}+Z_{ci4}=-\frac{2}{jwC_{i}}+Z_{1}+Z_{2}+Z_{3}$$

*w* is the angular frequency of the excitation voltage, and $Z_{Ci}$ is the equivalent capacitive impedance of the insulating layer [30]. $I_{0}$ can be expressed as

(4) $$I_{0}=\frac{V_{in}}{Z_{total}}$$

(5) $$V_{d}=V_{in}\cdot \frac{Z_{2}}{Z_{total}}$$

$I_{0}$ is the output current of the current output electrode $\left(E_{4}\right)$, and $V_{d}$ is the differential voltage between the voltage output electrodes ($E_{2}$ and $E_{3}$). The expression of the output signal can be expressed as

(6) $$\frac{I_{0}}{V_{d}}=\frac{V_{in}}{Z_{total}}/\frac{V_{in}Z_{2}}{Z_{total}}=\frac{1}{Z_{2}}=\frac{1}{R_{2}}+jwC_{2}=G_{2}+jwC_{2}$$

$G_{2}$ is the conductivity of the solution to be measured between the two voltage output electrodes ($E_{2}$ and $E_{3}$).

From Equation (6), it can be derived that in the five-electrode contactless conductivity detection method, the output signal depends on the solution conductivity ($G_{2}$) and the solution capacitance $\left(C_{2}\right)$. In general, $C_{2}$ << $G_{2}$, so its impact on detection can be approximately ignored. Compared with the traditional two-electrode contactless conductivity detection method, this five-electrode method effectively avoids the influence of the equivalent capacitive impedance of the insulating layer on detection. The five-electrode contactless conductivity detector thus operates effectively without requiring high excitation frequency. The use of a relatively lower operating frequency additionally reduces impedance contributions from both solution and inter-electrode coupling capacitances [32], providing better detection sensitivity. 

The operating principle and functional roles of the five-electrode contactless conductivity detector have been previously described. These electrodes work in concert to enable conductivity measurements through their coordinated operation. A sinusoidal AC voltage excitation signal is applied to the excitation electrode $\left(E_{1}\right)$. Through AC coupling between the insulating layer and the solution to be tested, a detection loop is formed between $E_{1}$ and $E_{4}$. The current output electrode $\left(E_{4}\right)$ outputs the current ${(I}_{0})$ flowing through the solution in the detection loop. Two voltage output electrodes $E_{2}$ and $E_{3})$ are placed between electrodes $E_{1}$ and $E_{4}$, and are externally connected to a voltage detection circuit with high input impedance, which results in almost zero current flowing through $E_{2}$ and $E_{3}$. Therefore, the voltage division effect of the insulating layer capacitor is small and can be ignored, permitting accurate measurement of the differential voltage $\left(V_{d}\right)$ between the two voltage output electrodes. A shielding electrode $\left(E_{5}\right)$ is arranged on the solution outlet side of the detection cell. Applying a shielding voltage raises the potential at the solution in the outlet $\left(E_{5}\right)$, which suppresses the leakage current in the flow channel of the detection unit and improves measurement accuracy.

### 2.3. Detection Dell Microchip Design and Fabrication

**Figure 2:**
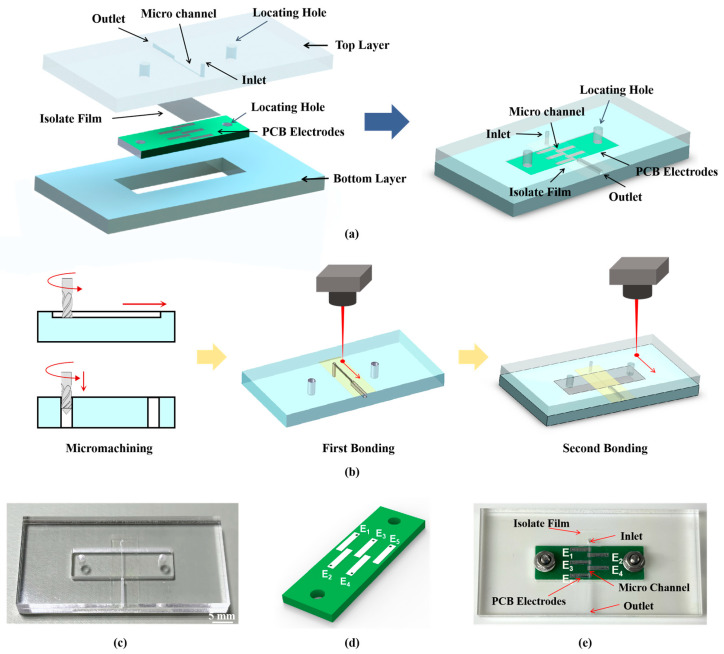
The design and fabrication of the sandwiched detection chip. (**a**) Sandwiched detection chip; (**b**) schematic diagram of the chip fabrication; (**c**) physical diagram of chip bonding, with dimensions of 50 mm × 25 mm × 6 mm (length × width × thickness); (**d**) physical diagram of PCB electrodes; (**e**) physical diagram of the sandwiched chip embedded with PCB electrodes.

Based on the above principle of five-electrode contactless conductivity detection, this study introduces a novel sandwiched detection chip with embedded printed circuit board (PCB) electrodes (Figure 2a). The overall size of the chip is 50 mm × 25 mm × 6 mm (length × width × thickness). This geometry was determined primarily by system integration requirements and fabrication feasibility.

To further optimize the detection performance, the detailed numerical simulations were conducted based on the Finite Element Method (FEM). Detailed simulation models and analysis results are provided in the Supplementary Materials (Figures S1 and S2). By systematically analyzing the impact of key structural parameters, such as microchannel dimensions, electrode spacing, and insulation layer properties, on the detection loop’s impedance and capacitive coupling efficiency, we determined the optimal internal geometry for the chip. The simulation results confirm that larger channel dimensions, optimized electrode spacing, and a thinner insulation layer with a higher dielectric constant significantly reduce total loop impedance and enhance sensitivity.

Based on the above simulation results, the chip was designed. The chip is composed of two poly(methyl methacrylate) (PMMA) layers and a polyvinyl chloride (PVC) film bonded together. The top PMMA layer incorporates a rectangular cross-section microchannel (8.3 mm × 400 μm × 300 μm), sample inlet/outlet ports, and alignment holes. The bottom PMMA layer contains a custom microfabricated region for PCB assembly, while a 50-μm-thick PVC film serves as a middle layer. On the PCB, the planar electrode array consists of five elements in sequential order: one excitation electrode, two voltage output electrodes, one current output electrode, and one shielding electrode. All electrodes maintain identical geometries of 5 mm × 1 mm × 35 μm (length × width × thickness), with inter-electrode spacings of 0.3 mm, 0.8 mm, 0.3 mm, and 0.8 mm. The PCB incorporates alignment holes along both edges for PCB integration.

Figure 2b illustrates the chip fabrication process. The PMMA layers with the microfabricated structure, such as a microfluidic channel and alignment holes, were fabricated by computer-numerical-control (CNC) micro-milling. The clearweld laser welding coatings were then uniformly coated on the bonding surface of the bottom PMMA layers. Two-stage laser bonding subsequently integrates the two PMMA layers with a polyvinyl chloride (PVC) film into a complete microchip (Figure 2c, presented without PCB). The contactless conductivity detection electrodes were fabricated as an integrated PCB module (Figure 2d) via a standardized industrial printed circuit board (PCB) manufacturing process, which included cutting, drilling, electroless plating, and chemical etching. Crucially, the geometry and space of all five electrodes were defined simultaneously using a single photolithographic mask to guarantee precise relative positioning and inter-electrode spacing. Finally, the PCB module was embedded in the bottom PMMA layer and secured via screw fixation to complete the assembly of the sandwiched chip (Figure 2e).

### 2.4. Detection Circuit Design

**Figure 3:**
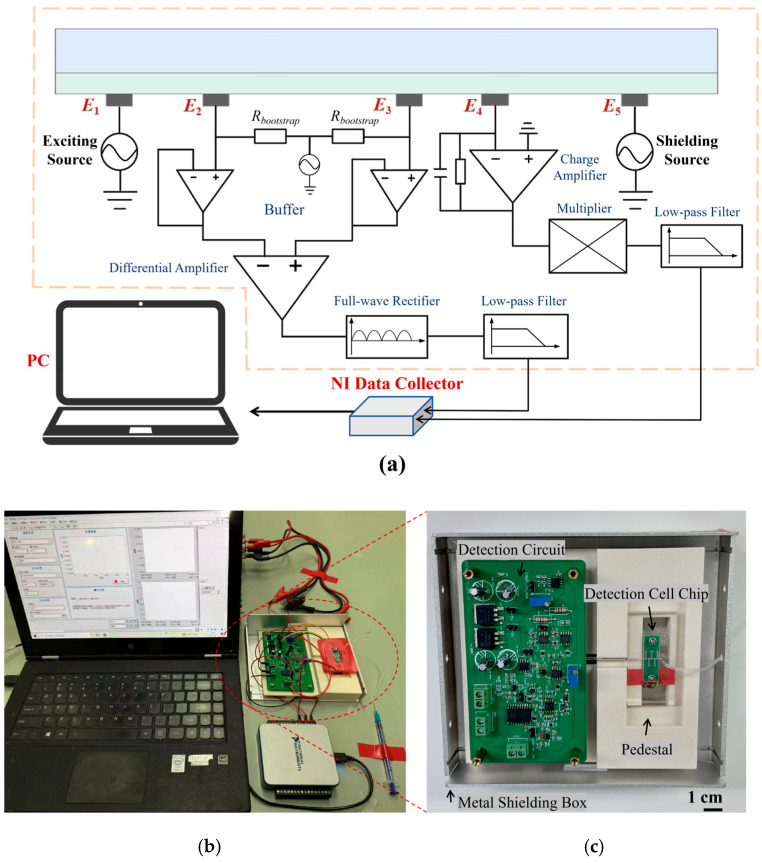
Schematics and integration diagrams of the five-electrode contactless conductivity detector. (**a**) Schematic diagram of the detection circuit; (**b**) integrated physical diagram of the detector; (**c**) physical diagram of the detector assembled, with dimensions of 155 mm × 148 mm (length × width).

Beyond the sandwiched detection chip, the circuit design is also critical for the detector, and mainly consists of two modules: a differential voltage detection module and a weak current detection module. The overall design scheme is shown in Figure 3a. The differential voltage detection module accurately measures the differential voltage across the sample solution between electrodes $E_{2}$ and $E_{3}$, and converts it into a DC signal for subsequent processing. Its core hardware incorporates three components: a high-input impedance circuit, an instrumentation amplifier circuit, and a rectification and filtering circuit. Specifically, the high-input impedance circuit presents a near-infinite input impedance to avoid voltage division across the insulating layer. The instrumentation amplifier integrates a buffer stage, thereby enhancing the common-mode rejection ratio (CMRR). The differential AC voltage signal is then full-wave rectified, and its peak value is extracted by a low-pass filter, resulting in a corresponding DC voltage signal. The other core module is the weak current detection module, which consists of a transimpedance amplifier (TIA), a phase-sensitive detector (PSD), and a low-pass filter (LPF). The TIA amplifies the sub-microampere-level current signal and converts it into a voltage output. The PSD performs a multiplication between the measured signal and a reference signal, while the LPF retains the DC component of the resultant signal. Finally, the detection circuit processes both output signals cooperatively. They are converted into a unified DC voltage signal, which facilitates subsequent data acquisition, analysis, and presentation.

### 2.5. Detector Assembly and Evaluation of Detector Performance

Figure 3b presents the integrated schematic of the five-electrode contactless conductivity detector. The two analog signals from the detection circuit were acquired by a host computer using a data acquisition card (USB-6001, National Instruments, Austin, TX, USA) for subsequent signal processing and presentation of the results. A dedicated data acquisition interface was developed to visualize the detected signals. To meet the experimental requirements for detector integration and stability, a custom 3D-printed base was designed and fabricated. Its structure is detailed in Figure 3c.

The detector’s performance was validated using three different standard solutions: NaCl, NaNO_3_, and Na_2_SO_4_ (concentration range of 0.1–140 ppm). The selection of these three distinct anions was based on their significance as critical indicators in public health and environmental monitoring [33,34,35]. These solutions were sequentially injected into the microchannel at a flow rate of 50 μL/min by a high-pressure constant-flow pump (N7100, Hanbang Technology, Nanjing, China). To ensure reproducibility, before each injection, the microchannel was flushed with deionized water for 2 min at 200 μL/min to remove residual ions and restore the signal baseline. Each solution was measured in triplicate, and subsequently, the linear regression coefficient was calculated. Finally, after the experiments, the chip was thoroughly rinsed with deionized water, dried, and stored in a clean environment at room temperature.

### 2.6. Detection After Chromatography Separation

To further assess the performance of the five-electrode contactless conductivity detector, an IC system was established based on prior work. The system components included a high-pressure constant-flow pump, a six-way valve injector valve, an anion chromatography column, a suppressor, a data acquisition card, and a host computer. For the analysis, the eluent, composed of 3 mM sodium bicarbonate and 5 mM sodium carbonate, was pumped into a commercially available six-way valve injector valve (Model 7725i-04, Rheodyne, Rohnert Park, CA, USA) using a high-pressure pump. This pump maintained a constant mobile phase flow rate of 100 µL/min. Concurrently, a 10 µL aliquot of the test sample was injected into the valve and then passed through an anion chromatography column (Model CA2, Qingdao Ruipu, Qingdao, China). Following on-column separation, a suppressor (WLK-10A, Qingdao Ruipu, Qingdao, China) was used to lower the background conductivity of the eluent in the separated products from the column. The separated components were then detected using the five-electrode contactless conductivity detector, which measured the corresponding changes in solution conductivity. Finally, the acquired data were transmitted to a handheld computer via a USB interface and smoothed by applying a digital filter.

## 3. Results and Discussion

### 3.1. Optimization of Conditions

**Figure 4:**
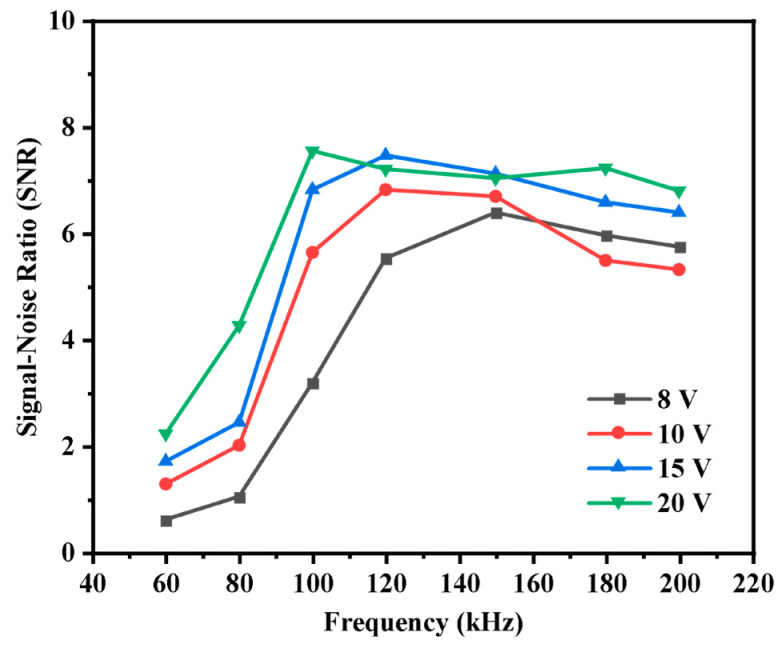
Effect of excitation signal amplitude and frequency on the SNR of the output signals.

The frequency and amplitude of the excitation signal have a significant impact on detector performance. To select the appropriate measurement conditions, the influence of different frequencies and amplitudes of the excitation signal on the signal-to-noise ratio (SNR) of detection signals was studied by recording signal responses of the detector to a fixed-concentration NaCl sample. Specifically, the amplitude of the excitation signal was set at 8 V, 10 V, 15 V, and 20 V, respectively. And the SNR of the detector signal was examined under different excitation frequencies (60 kHz, 80 kHz, 120 kHz, 150 kHz, and 200 kHz). Figure 4 shows the effects of excitation parameters on the SNR of the detector signal. It is obvious that the SNR significantly increases as the frequency of the excitation signal increases from 60 kHz to 120 kHz, when the amplitude of the excitation signal was fixed at 8 V, 10 V, and 15 V, respectively. As the excitation frequency exceeds 120 kHz, the SNR reaches a maximum and subsequently decreases. This phenomenon is attributed to the inherent conflict between minimizing the insulating layer’s impedance and suppressing parasitic capacitive noise [36]. Although increasing the frequency can initially mitigate the impact of the insulating layer on the output signal by reducing the insulating layer’s capacitive reactance ($Z_{ci}$), the influence of solution dielectric effects and stray capacitance coupled through the air will be exacerbated when frequency exceeds 100 kHz. This leads to a significant rise in the system noise, which compromises detection accuracy and results in poorer linear response. When the frequency of the excitation signal was set below 100 kHz, there is a significant upward trend for SNR as amplitude of the excitation signal increased from 8 V to 20 V, while, in the frequency of 120 or 140 kHz, the higher SNR was obtained with the amplitude of 15 V. Considering practical constraints, the five-electrode contactless conductivity detector was ultimately operated with a 15 V, 120 kHz excitation signal. Under these conditions, the detector achieved an SNR of 7.5 for 5 ppm NaCl samples. While the SNR is lower than that of traditional contact conductivity detectors, it is comparable with the typical range (1–20) reported for commercial contactless detectors [23]. Furthermore, to further enhance the SNR for practical application, digital filtering was employed.

In addition to the frequency and amplitude of the excitation signal, the selection of the shielding voltage amplitude is also critical. Leakage current is influenced by multiple complex factors, such as the detection cell’s geometry and ambient conditions, making it difficult to determine the optimal shielding amplitude through simulation alone. To select the appropriate suitable shielding conditions, we evaluated detector linearity using NaCl standard solutions (5–50 ppm) and examined how different shielding amplitudes affect linear response. All measurements were conducted under constant excitation conditions of 15 V and 120 kHz. The shielding signal amplitude was systematically varied across the values of 0, 0.5, 0.8, 1.0, 1.2, 1.5, and 2.0 V. For each shielding amplitude, the linear response was evaluated by performing triplicate measurements across the full concentration range. Subsequently, a calibration curve was constructed via linear regression analysis of the detector output versus NaCl concentration, from which the coefficient of determination (R^2^) was derived. These calculated R^2^ values are summarized in Table S1. The results indicate that as the shielding amplitude increases, the response linearity initially improves, followed by a decline. Within the range of 0 V to 1.2 V, the suppression of leakage current is enhanced as the amplitude increases. However, in the range of 1.2 V to 2.0 V, excessive shielding voltage leads to current over-compensation, thereby deteriorating performance. The optimal shielding parameters are identified as an amplitude of 1.2 V and a frequency of 120 kHz. Under these optimized conditions, the R^2^ for NaCl standard solutions exceeds 0.996.

### 3.2. Baseline Noise and SNR Measurements

Noise is a critical parameter governing detector performance, as its magnitude directly influences detection accuracy and the limit of detection (LOD). This study evaluates detector performance by measuring baseline noise and the signal-to-noise ratio. Baseline noise corresponds to the signal fluctuation observed in the absence of analyte within the detection cell. The standard deviation is conventionally employed to quantify this noise. For a signal comprising N data points (where the i-th point is denoted as $X_{i}$), the noise $\left(X_{SD}\right)$ can be expressed as

(7) $$X_{SD}=\sqrt{\frac{1}{N}\sum_{1}^{N}{\left(X_{i}-\frac{1}{N}\sum_{1}^{N}X_{i}\right)}^{2}}$$

**Figure 5:**
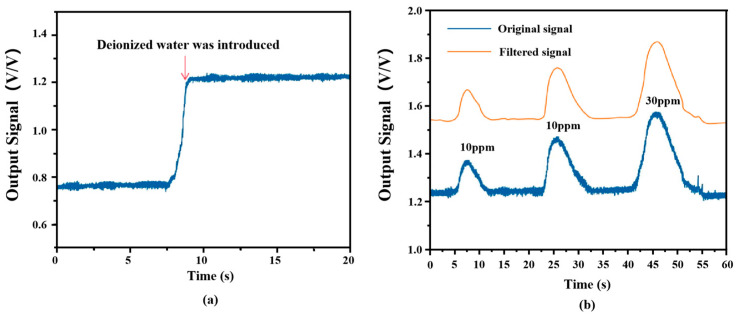
Noise level measurement results. (**a**) Detector baseline noise signal; (**b**) comparison between original signal data (blue) and signal data after digital filtering (orange).

As illustrated in Figure 5a, deionized water was pumped through the microchannel at a constant flow rate of 50 µL/min. Upon reaching the detection zone, the system exhibited a characteristic signal transition. The standard deviation of the recorded signal was subsequently computed to determine the baseline noise, resulting in a measured raw baseline noise value of 1.44 × 10^−2^ V/V for the detector.

Digital filtering is considered an effective technique for noise reduction [37]. In this study, post-processing was performed to mitigate noise interference. Specifically, a sliding window average filtering algorithm was applied to the acquired raw signals. This method functions by calculating the average of data points within a moving window along the time series, thereby smoothing out high-frequency random noise while retaining the low-frequency signal components. As shown in Figure 5b, digital filtering preserves the original shape and intensity of the chromatographic peaks. The signal-to-noise ratios (SNRs) for NaCl standard solutions at varying concentrations (10, 20, and 30 ppm) demonstrate significant enhancement after filtering. The SNRs increased from 8.54, 14.69, and 22.43 (unfiltered) to 39.71, 99.89, and 152.52, respectively. The smoothness of the curve is improved, and the integrity of the original data regarding peak height and area values is preserved by the filtering. The above enhancement can significantly reduce noise interference, thereby facilitating the process for subsequent data analysis.

### 3.3. Linearity Measurements and LOD

**Figure 6:**
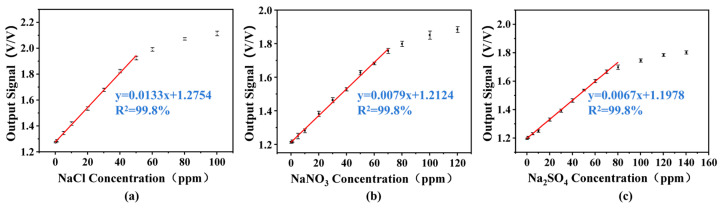
Linear relationship between detector output signal and sample concentration. (**a**) NaCl; (**b**) NaNO_3_; (**c**) Na_2_SO_4_.

To further analyze the performance of the detection system, the linearity and sensitivity of the detector are studied. Standard solutions of NaCl, NaNO_3_ and Na_2_SO_4_ with different concentrations were used as detection samples. A high-pressure constant-flow pump was used to successively introduce the sample solution into the microchannel of the chip at a flow rate of 50 µL/min. The detector responses to standard solutions at varying concentrations were recorded three times. All measurements were performed sequentially in order of increasing concentration, with a deionized water rinse step implemented between each analysis. Figure 6 presents the response values of the detector to each sample with different concentrations, obtained under optimized conditions (sinusoidal excitation signal: 15 V, 120 kHz; shielding voltage: 1.2 V, 120 kHz). The linear fitting was performed, and the linear response correlation coefficient R^2^ was calculated. It is obvious that calibration curves for NaCl, NaNO_3_, and Na_2_SO_4_ all exhibit high linearity because the linear correlation coefficient R^2^ for the three samples all reached 0.998. Specifically, as shown in Figure 6a, the detector has a good linear response to NaCl samples within the concentration range of 0.1 ppm to 50 ppm. The detector exhibited a sensitivity of 1.33 × 10^−2^ V·(V·ppm)^−1^ for NaCl quantification within its linear range. Moreover, this five-electrode contactless conductivity detector also shows excellent linear response characteristics for both NaNO_3_ and Na_2_SO_4_ samples and the linear ranges were from 0.1 to 60 ppm for the NaNO_3_ sample and from 0.1 to 70 ppm for the Na_2_SO_4_ sample, respectively. The variation in detection sensitivity between these analytes was less than 10%, with both linear correlation coefficients (R^2^) of 0.998. Within their respective linear ranges, the sensitivities of the detector’s output signals were 7.9 × 10^−3^ V·(V·ppm)^−1^ and 6.7 × 10^−3^ V·(V·ppm)^−1^ for NaNO_3_ and Na_2_SO_4_ samples, respectively. Such linear responses align well with the expectation that the detector’s output signal is positively correlated with the concentration of the analyte.

The LOD, determined by the response value at three times the detector’s baseline noise level, serves as a metric reflecting the minimum detection capability of an analytical instrument [35]. It can be expressed as

(8) $$D=\frac{3X_{SD}}{S}$$

D is the minimum detection limit of the detector, $X_{SD}$ is the baseline noise level, and S is the sensitivity of the detector. 

From formula (8), the LOD values of the five-electrode contactless conductivity detector in this paper for Cl^−^, NO_3_^−^, and SO_4_^2−^ are calculated to be 0.47 ppm, 0.80 ppm, and 0.95 ppm, corresponding to a concentration range of approximately 10–13 μM, as summarized in Table 1. This value is smaller than that (50–100 μM) of the planar grounded capacitively coupled contactless conductivity detector (PG-C^4^D) with two-electrode configuration reported by Wang et al. [38]. The reason is that the contactless conductivity detector with five-electrode configuration developed in this work achieves effective decoupling of the excitation and measurement loops and reduces leakage current. In addition, the LOD for this contactless conductivity detector developed is comparable to that of the Poly(dimethylsiloxane) C^4^D developed by Koczka et al. [39]. Moreover, the electrode PCB module is embedded within the detection chip in this study, eliminating these manual operation steps for each detection, thereby significantly enhancing the structural stability and operational convenience of the detection device.

### 3.4. IC System Separation and Detection

**Figure 7:**
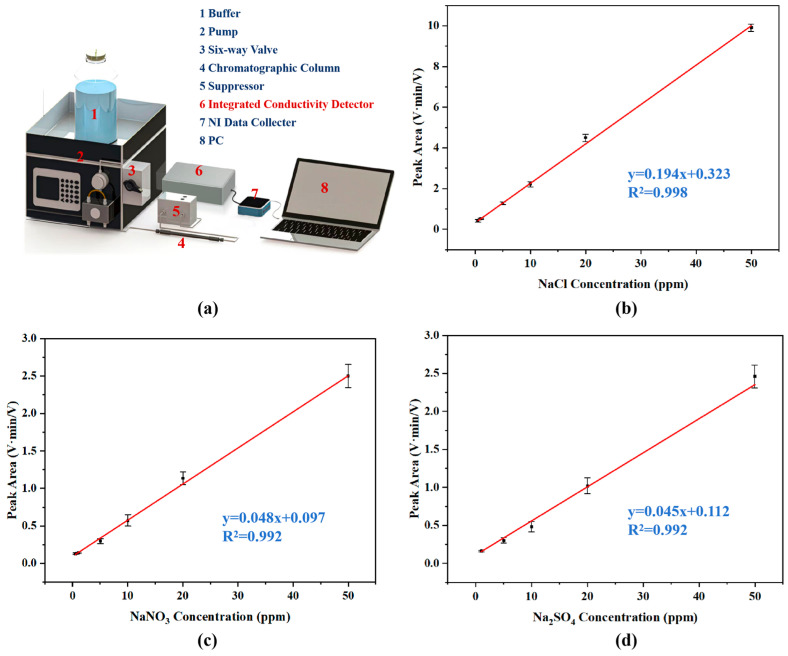
Accuracy and repeatability results after chromatographic separation. (**a**) Schematic diagram of the ion chromatography system; (**b**) relationship between detector output signal and NaCl sample concentration; (**c**) relationship between detector output signal and NaNO_3_ sample concentration; (**d**) relationship between detector output signal and Na_2_SO_4_ sample concentration.

To test the detection performance of the five-electrode contactless conductivity detector in the IC system in this study, an IC separation and detection system for the separation and detection of mixed anion samples was built based on the basic principles of IC (Figure 7a). To reduce the background conductivity of the eluent, a WLK-10A suppressor was applied. Subsequently, the five-electrode contactless conductivity detector monitored the resultant conductivity variations, confirming its detection capability for post-separation ionic species in the IC system. Each individual standard solution, such as NaCl, NaNO_3_, or Na_2_SO_4_, was tested three times by the above ion chromatography separation and detection system, and the corresponding results are presented in Table 2. Figure 7b–d illustrates the response of the detector within the ion chromatography system, which is closely correlated with the intrinsic response characteristics to standard solutions demonstrated in Figure 6. The high sensitivity established in Figure 6 serves as the prerequisite for the detector to achieve the successful detection of separated substances in the ion chromatography system.

It is obvious that the five-electrode contactless conductivity detector exhibits strong linear responses to Cl^−^, NO_3_^−^, and SO_4_^2−^ over defined concentration ranges, and the corresponding linear correlation coefficients (R^2^) are 0.998, 0.998, and 0.997, respectively. These results demonstrate excellent linear response characteristics across specified concentration ranges for all three anions. Additionally, the separation system also demonstrated high reproducibility in retention times across individual ion species. The relative standard deviations (RSDs) for Cl^−^, NO_3_^−^, and SO_4_^2−^ were measured at 2.0%, 3.4%, and 1.9%, respectively. These findings confirm that the contactless conductivity detector developed in this study can enable qualitative and quantitative analysis of the above three anions within a certain concentration range in IC systems.

**Figure 8:**
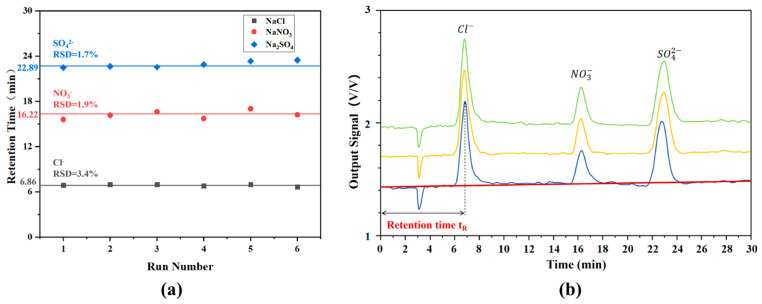
Response curves of the ion chromatography conductivity detection system for mixed samples. (**a**) Retention time repeatability; (**b**) chromatographic separation curves of the three anions.

To further evaluate the performance of the five-electrode contactless conductivity detector in the IC system, a mixed solution containing NaCl (5 ppm), NaNO_3_ (10 ppm), and Na_2_SO_4_ (20 ppm) was analyzed, and the results are presented in Figure 8. Figure 8a illustrates the retention time profiles of different ions (Cl^−^, NO_3_^−^, and SO_4_^2−^) across multiple experiments. The mean retention time for Cl^−^ was 6.86 min with a relative standard deviation (RSD) of 3.4%; for NO_3_^−^, the mean retention time was 16.22 min with an RSD of 1.9%; and for SO_4_^2−^, the mean retention time was 22.89 min with an RSD of 1.7%. The relatively small RSD values for retention times indicate that the IC conductivity detection system exhibits good repeatability in retention times for different ions during 6 runs. Chromatographic peak identification was achieved by matching the retention times with those of individual standard solutions, confirming the three peaks as Cl^−^, NO_3_^−^, and SO_4_^2−^. The relative standard deviations (RSD, n = 3) for the peak areas of these three components were calculated as 1.3%, 2.2%, and 1.9%, respectively. The ion concentrations in the mixed solution were quantified by referencing the fitting straight line of each single component’s linear response data in Figure 7. The analytical results for the separated mixture revealed measured concentrations of 5.03 ppm, 4.94 ppm, and 5.11 ppm for Cl^−^ (maximum detection error of 2.2%); 10.12 ppm, 9.85 ppm, and 9.92 ppm for NO_3_^−^ (maximum detection error of 1.5%); and 20.32 ppm, 20.15 ppm, and 20.08 ppm for SO_4_^2−^ (maximum detection error of 1.6%), which is better than the detector proposed by Adam J et al. [40], and comparable to the performance of a conventional commercial instrument [41]. These low error values demonstrate high consistency between the mixed and individual solution scenarios, confirming that the detector’s response characteristics established with individual solutions remain valid for complex mixtures. As shown in the chromatogram (Figure 8b), the anion exchange column effectively separates Cl^−^, NO_3_^−^, and SO_4_^2−^ into distinct, non-overlapping peaks. This baseline separation ensures that when a specific anion passes through the detector, other anions are absent, thereby avoiding signal crosstalk or superposition. Furthermore, the baseline stability during the continuous separation process was quantitatively analyzed. Although a slight baseline fluctuation is observable in Figure 8b, a linear fit of the baseline signal yielded a slope of only1.74 × 10^−3^ V/V·min, indicating minimal drift. This phenomenon is likely attributed to (1) temperature-induced conductivity variations; (2) trace contamination from sample residues; and (3) inherent electronic drift in the detection system [42]. However, this drift is negligible relative to the peak signals and does not compromise the integration accuracy. Consequently, these results demonstrate the capability of the five-electrode contactless conductivity detector for the identification and quantification of multiple ionic analytes in IC applications.

## 4. Conclusions

Herein, a miniaturized chip-based five-electrode contactless conductivity detector has been developed for miniaturized IC detection. This detector comprises two main components: a detection chip and its associated detection circuit. The chip employs a sandwich structure consisting of a top layer, an insulating film, and a bottom layer that embeds a planar five-electrode PCB. Among the five electrodes, the introduced shielding electrode effectively reduces the leakage current in the flow channel of the detection chip. Moreover, the five electrodes are designed and fabricated as a PCB module, which can simplify the fabrication process and enhance the lifespan of the electrodes. The application within a commercial IC system to detect NaCl, NaNO_3_, and Na_2_SO_4_ standard solutions showed the detector exhibited a strong linear response, achieving linear correlation coefficients (R^2^) of no less than 0.998. In IC analyses of mixed samples, the system successfully separated and quantified multiple ionic species with maximum detection errors below 2.2%. These results demonstrate that the chip-based five-electrode contactless conductivity detector has great potential for application in miniaturized ion chromatography. Currently, the detector is coupled with a commercial chromatographic column to validate its fundamental performance. In future work, upon achieving full system integration with other miniaturized components, we will extend the evaluation to real environmental samples to validate the practical application potential of the fully miniaturized ion chromatography system.

## Figures and Tables

**Table 1:** Limit of Detection (LOD) of the Five-Electrode contactless conductivity detector and comparison of the miniaturized detector in this study with others.

Detector	Detection Limit (LOD)	References
PG-C^4^D	50–100 μM	[38]
PDMS chip-C^4^D	3.66–14.7 μM	[39]
Five-Electrode C^4^D	10–13 μM	This work

**Table 2:** Data from peak areas and retention times at different concentrations.

Ions	Concentration (ppm)	0.5	1	5	10	20	50
**Cl^−^**	Average Peak Area (V·min/V)	0.42	0.52	1.29	2.24	4.49	9.92
	Average Retention Time (min)	6.58	6.69	6.88	6.79	6.91	6.65
**NO_3_^−^**	Average Peak Area (V·min/V)	0.13	0.14	0.30	0.59	1.12	2.48
	Average Retention Time (min)	15.50	16.35	16.84	16.62	17.15	16.57
**SO_4_^2−^**	Average Peak Area (V·min/V)	-	0.16	0.28	0.48	1.03	2.55
	Average Retention Time (min)	-	22.08	22.55	22.89	22.33	23.15

## Data Availability

The raw data supporting the conclusions of this article will be made available by the authors on request.

## Supplementary Materials

The following supporting information can be downloaded at: https://www.mdpi.com/article/10.3390/s26010089/s1, Figure S1: Schematic diagram of the 3D simulation model for the five-electrode contactless conductivity detection cell; Figure S2: Effect of various structural parameters on the admittance of the detection circuit; Figure S3: Scanning electron microscopy (SEM) images of the PCB modules with the five-electrodes; Table S1: Detector linear response under different shielding signal amplitudes; Table S2: Simulation calculation results of solution resistance (R) parameters.
