# Supplementary material for: A Five-Electrode Contactless Conductivity Detector Based on a Sandwiched Microfluidic Chip for Miniaturized Ion Chromatography
Source: Sensors (Basel). 2025 Dec 23;26(1):89. doi: 10.3390/s26010089 (PMC12788068; doi:10.3390/s26010089)
Supplement: Supplementary file 1 [file sensors-26-00089-s001.zip › sensors-3991519-supplementary.pdf]

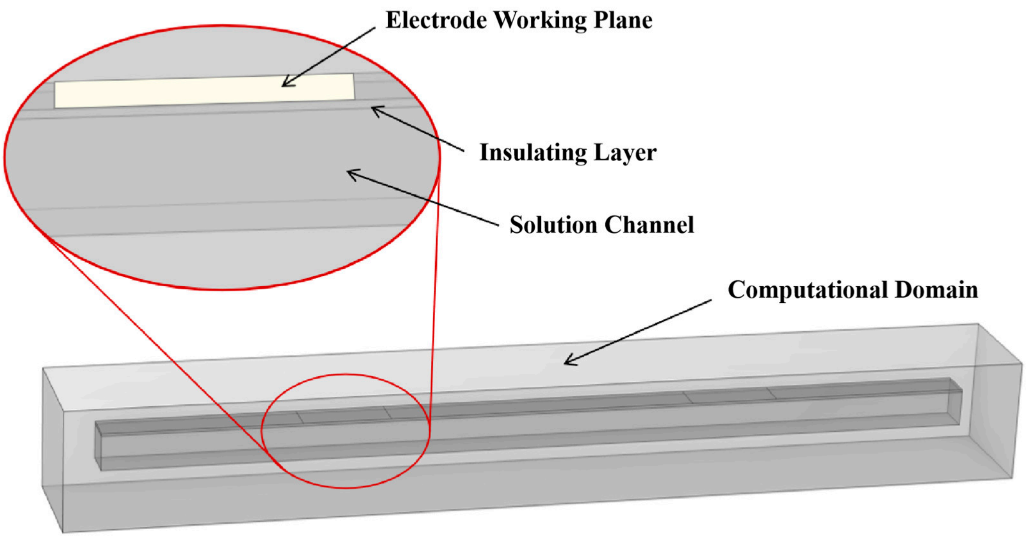

Figure S1. Schematic diagram of the 3D simulation model for the five-electrode contactless conductivity detection cell.

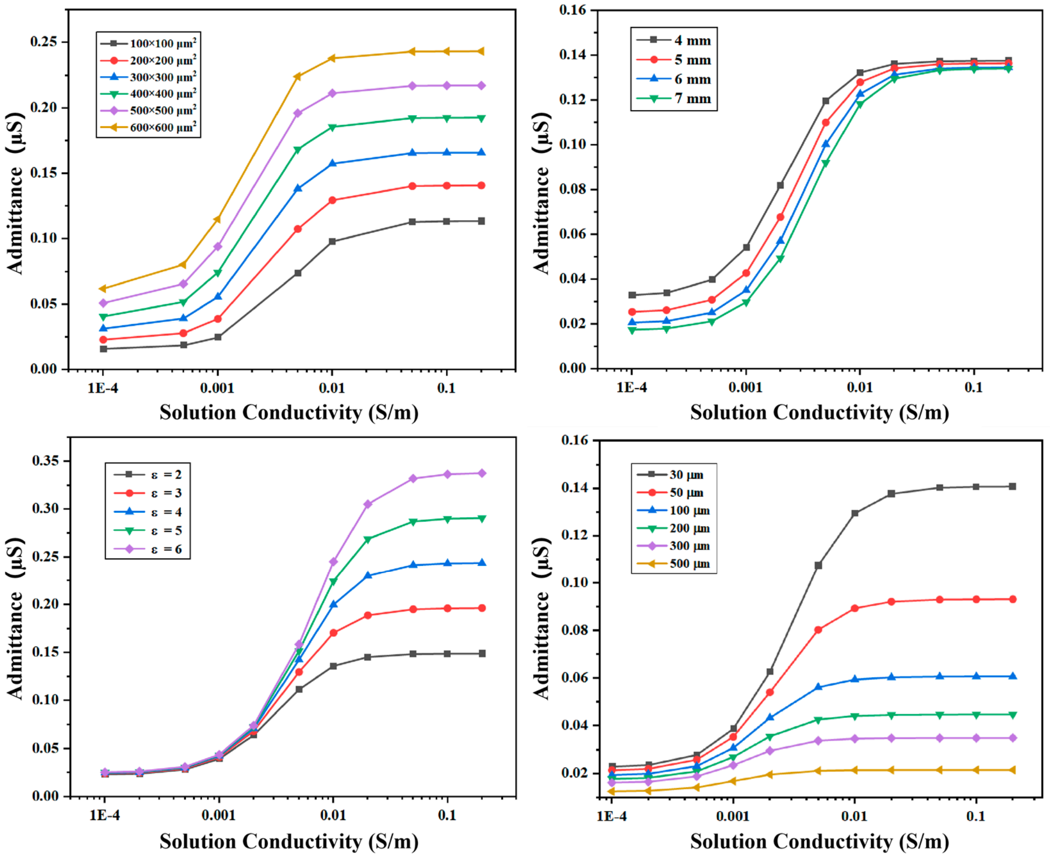

Figure S2. Effect of various structural parameters on the admittance of the detection circuit. (**a**) Channel dimensions of the detection cell; (**b**) electrode spacing; (**c**) relative permittivity of the insulation layer material; (**d**) thickness of the insulation layer.

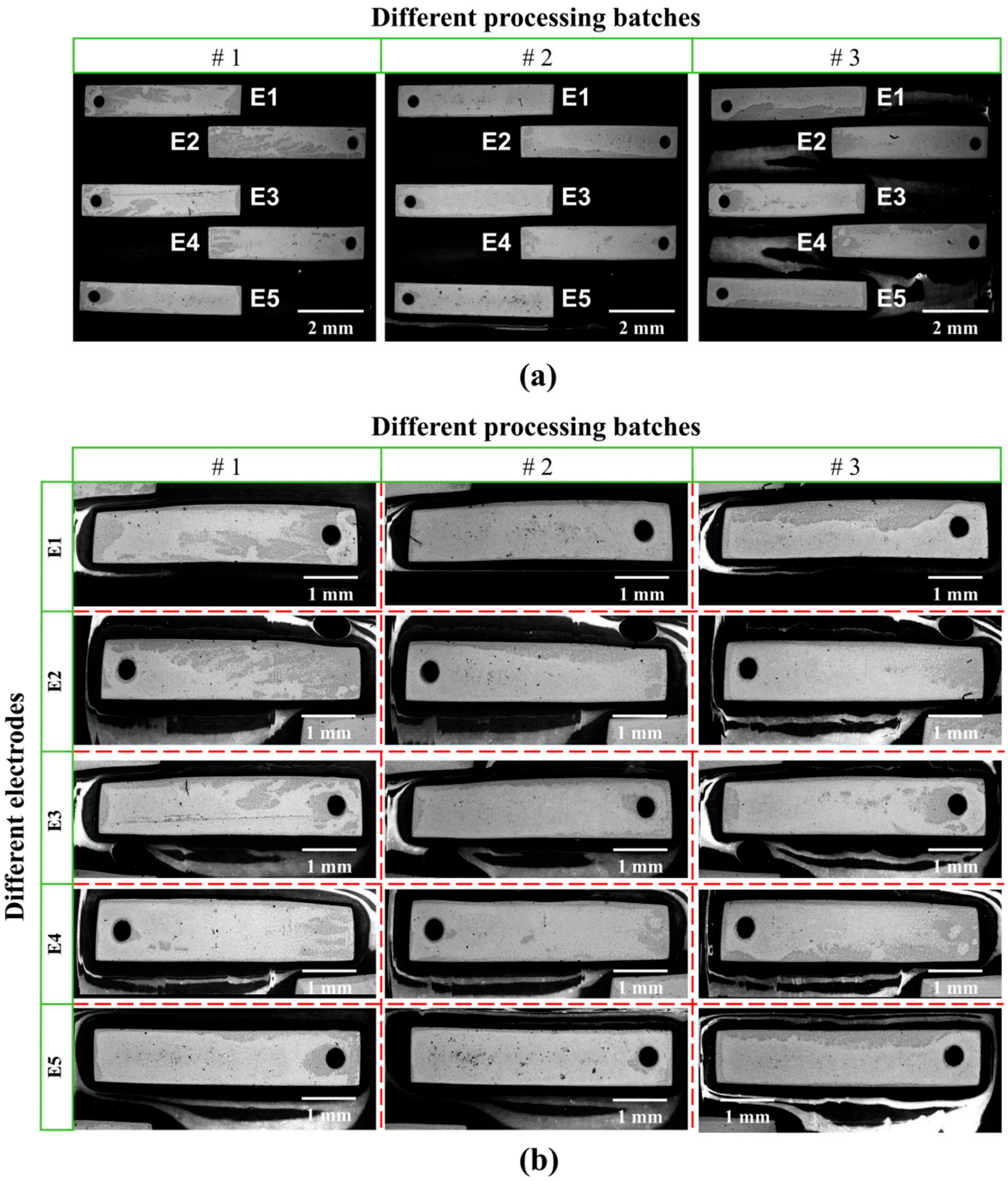

Figure S3. Scanning electron microscopy (SEM) images of the PCB modules with the five-electrodes. (a) Different processing batches; (b) different electrodes.

Table S1. Detector linear response under different shielding signal amplitudes.

amplitudes / R^2 / test	0 V	0.5 V	0.8 V	1.0 V	1.2 V	1.5 V	2.0 V
**#1**	0.9885	0.9924	0.9934	0.9958	0.9968	0.9957	0.9899
**#2**	0.9912	0.9907	0.9928	0.9935	0.9962	0.9960	0.9916
**#3**	0.9906	0.9939	0.9937	0.9952	0.9961	0.9954	0.9923

Table S2. Simulation calculation results of solution resistance (R) parameters.

L (mm) / R (Ω) / C(mM)	0.01 V	0.1 V	1.0 V	5.0 V
**500**	3.74×10^5	3.74×10^4	3.74×10^3	6.89×10^2
**700**	5.21×10^5	5.21×10^4	3.74×10^3	8.26×10^2
**1000**	6.88×10^5	6.88×10^4	6.88×10^3	14.2
